# Development of a Prototype Lateral Flow Immunoassay (LFI) for the Rapid Diagnosis of Melioidosis

**DOI:** 10.1371/journal.pntd.0002727

**Published:** 2014-03-20

**Authors:** Raymond L. Houghton, Dana E. Reed, Mark A. Hubbard, Michael J. Dillon, Hongjing Chen, Bart J. Currie, Mark Mayo, Derek S. Sarovich, Vanessa Theobald, Direk Limmathurotsakul, Gumphol Wongsuvan, Narisara Chantratita, Sharon J. Peacock, Alex R. Hoffmaster, Brea Duval, Paul J. Brett, Mary N. Burtnick, David P. AuCoin

**Affiliations:** 1 InBios International, Inc., Seattle, Washington, United States of America; 2 Department of Microbiology and Immunology, University of Nevada School of Medicine, Reno, Nevada, United States of America; 3 Menzies School of Health Research and Northern Territory Clinical School, Royal Darwin Hospital, Darwin, Northern Territory, Australia; 4 Department of Tropical Hygiene, Mahidol-Oxford Tropical Medicine Research Unit, Faculty of Tropical Medicine, Mahidol University, Bangkok, Thailand; 5 Department of Clinical Pathology, Sappasithiprasong Hospital, Ubon Ratchathani, Thailand; 6 Department of Microbiology and Immunology, and Mahidol-Oxford Tropical Medicine Research Unit, Faculty of Tropical Medicine, Mahidol University, Bangkok, Thailand; 7 Mahidol-Oxford Tropical Medicine Research Unit, Faculty of Tropical Medicine, Department of Microbiology and Immunology, Mahidol University, Bangkok, Thailand, and Department of Medicine, Cambridge University, Addenbrooke's Hospital, Cambridge, United Kingdom; 8 Bacterial Special Pathogens Branch, Division of High-Consequence Pathogens and Pathology, National Center for Emerging and Zoonotic Infectious Diseases, Centers for Disease Control and Prevention, Atlanta, Georgia, United States of America; 9 Department of Microbiology and Immunology, University of South Alabama, Mobile, Alabama, United States of America; Institut Pasteur, France

## Abstract

*Burkholderia pseudomallei* is a soil-dwelling bacterium and the causative agent of melioidosis. Isolation of *B. pseudomallei* from clinical samples is the “gold standard” for the diagnosis of melioidosis; results can take 3–7 days to produce. Alternatively, antibody-based tests have low specificity due to a high percentage of seropositive individuals in endemic areas. There is a clear need to develop a rapid point-of-care antigen detection assay for the diagnosis of melioidosis. Previously, we employed In vivo Microbial Antigen Discovery (InMAD) to identify potential *B. pseudomallei* diagnostic biomarkers. The *B. pseudomallei* capsular polysaccharide (CPS) and numerous protein antigens were identified as potential candidates. Here, we describe the development of a diagnostic immunoassay based on the detection of CPS. Following production of a CPS-specific monoclonal antibody (mAb), an antigen-capture immunoassay was developed to determine the concentration of CPS within a panel of melioidosis patient serum and urine samples. The same mAb was used to produce a prototype Active Melioidosis Detect Lateral Flow Immunoassay (AMD LFI); the limit of detection of the LFI for CPS is comparable to the antigen-capture immunoassay (∼0.2 ng/ml). The analytical reactivity (inclusivity) of the AMD LFI was 98.7% (76/77) when tested against a large panel of *B. pseudomallei* isolates. Analytical specificity (cross-reactivity) testing determined that 97.2% of *B. pseudomallei* near neighbor species (35/36) were not reactive. The non-reactive *B. pseudomallei* strain and the reactive near neighbor strain can be explained through genetic sequence analysis. Importantly, we show the AMD LFI is capable of detecting CPS in a variety of patient samples. The LFI is currently being evaluated in Thailand and Australia; the focus is to optimize and validate testing procedures on melioidosis patient samples prior to initiation of a large, multisite pre-clinical evaluation.

## Author Summary

*Burkholderia pseudomallei* is an environmental bacterium and the cause of melioidosis. Culture of patient samples is the “gold standard” diagnostic test, but may take up to 7 days to complete. Melioidosis has a 10–40% case fatality rate depending on the geographic location. Delays in diagnosis could lead to administration of ineffective antimicrobial therapy, since *B. pseudomallei* is resistant to empiric antibiotic regimens. Therefore, we have developed a lateral flow immunoassay that can be used in the clinical setting to diagnose melioidosis in 15 minutes. The test promises to provide improved management of patients with melioidosis.

## Introduction

*Burkholderia pseudomallei* is an environmental Gram-negative bacillus and the cause of melioidosis. The clinical manifestations of melioidosis are broad and include disseminated disease with organ abscesses, severe sepsis, and mild infection of the skin and soft tissue [1]. Most patients have risk factors for infection, which include diabetes, heavy alcohol use, and chronic pulmonary or kidney disease [1]–[3]. The highest number of reported cases occurs in endemic regions of Thailand and Australia. Rising incidence rates have been recorded in northeast Thailand between 1997–2006, during which the average mortality rate was 42.6% [3]. In 2006, melioidosis and tuberculosis mortality rates in northeast Thailand were equivalent and second only to HIV/AIDS for infectious disease deaths [3]. In northern Australia the mortality rate over the last five years of the Darwin prospective melioidosis study was calculated at 9% [2]. The authors attributed the low mortality rate to early diagnosis and treatment, and access to and improvements in intensive care management [2].

Isolation of *B. pseudomallei* from clinical samples remains the “gold standard” against which other melioidosis diagnostics are compared [4]. Culture is routinely performed on multiple sample types (blood, urine, pus, sputum, etc.) and isolation of *B. pseudomallei* from any one of these cultures is diagnostic for melioidosis [5], [6]. However, recent modeling data has confirmed that culturing is an imperfect gold standard [7]. Furthermore, laboratory processing of positive samples takes 3–7 days [8]. This problem is compounded by the fact that many diagnostic laboratories may misidentify *B. pseudomallei* through lack of experience or validated diagnostic reagents [9]. Any delay in diagnostic confirmation is potentially important as *B. pseudomallei* requires therapy with ceftazidime or a carbapenem drug, which are not agents of choice for empirical therapeutic regimens. Taken together, these factors point to a clear need for a simple and rapid diagnostic test for accurate identification of *B. pseudomallei* directly on clinical samples or cultures.

Prior to diagnostic test development we identified a number of potential *B. pseudomallei* diagnostic biomarkers by *In vivo* Microbial Antigen Discovery (InMAD) [10], [11] that are shed or secreted and may be targeted to diagnose acute disease. Capsular polysaccharide (CPS) proved to be the most encouraging target; this molecule is a polymer of 1,3-linked 2-*O*-acetyl-6-deoxy-β-d-*manno*-heptopyranose residues [12]. We confirmed CPS was present in melioidosis patient serum and urine samples by antigen-capture ELISA utilizing a CPS-specific monoclonal antibody (mAb 3C5) [10]. The current report describes the characterization of mAb 3C5, quantification of CPS within patient samples, and optimization of the Active Melioidosis Detect lateral flow immunoassay (AMD LFI) for the rapid diagnosis of melioidosis.

## Materials and Methods

### Bacterial cultures

Bacterial isolates listed in Table 1 were cultured on trypticase soy agar containing 5% sheep blood. *Escherichia coli* and *B. pseudomallei* (strain Bp82) were cultured on Luria Bertani agar and brain heart infusion agar, respectively. Plates were incubated at 37°C for 18–24 h. All work with viable *B. pseudomallei* and *Burkholderia mallei* strains was conducted under BSL-3 containment. All other strains were grown under BSL-2 containment.

**Table 1:** Active Melioidosis Detect analytical reactivity and specificity.

Bacterial isolate	Strain name/DASH #	Lateral Flow Result
*Burkholderia pseudomallei*	7641; PHLS24; CDC2721620	Positive (+)
*Burkholderia pseudomallei*	Bp25; CDC2721628; 770429	Positive (+)
*Burkholderia pseudomallei*	CDC2721639; PHLS 66	Positive (+)
*Burkholderia pseudomallei*	K96243; NR 9320; CDC0022138	Positive (+)
*Burkholderia pseudomallei*	Bp92; CDC2721623	Positive (+)
*Burkholderia pseudomallei*	Thai 2 NE Human 88; PHLS 45	Positive (+)
*Burkholderia pseudomallei*	Bp104; CDC2721624	Positive (+)
*Burkholderia pseudomallei*	CDC2721635; PHLS 36	Positive (+)
*Burkholderia pseudomallei*	Bp73; Ln31348	Positive (+)
*Burkholderia pseudomallei*	PHLS 208	Positive (+)
*Burkholderia pseudomallei*	CDC2721102; F5013	Positive (+)
*Burkholderia pseudomallei*	BpG9709; CDC0032026	Positive (+)
*Burkholderia pseudomallei*	Sing Env 91; PHLS 19; CDC2721625	Positive (+)
*Burkholderia pseudomallei*	ATCC 23343; CDC2721676; NCTC 12939	Positive (+)
*Burkholderia pseudomallei*	Bp2889; SID2889	Positive (+)
*Burkholderia pseudomallei*	France Env 76; PHLS 33; CDC2721630; 7605	Positive (+)
*Burkholderia pseudomallei*	Bp68; CDC2721641	Positive (+)
*Burkholderia pseudomallei*	Indo 1 Monkey 90; PHLS 17; CDC2721619	Positive (+)
*Burkholderia pseudomallei*	Sing3 Human 88; PHLS 38; S6	Positive (+)
*Burkholderia pseudomallei*	1106a; U1106a; CDC0022030	Positive (+)
*Burkholderia pseudomallei*	Bp53; CDC2721633; 307a	Positive (+)
*Burkholderia pseudomallei*	Bp24; CDC2721620	Positive (+)
*Burkholderia pseudomallei*	BpG9313; CDC0032029	Positive (+)
*Burkholderia pseudomallei*	CDC2721162; B7210; B6195; 904-1111	Positive (+)
*Burkholderia pseudomallei*	CDC2721114; G6715	Positive (+)
*Burkholderia pseudomallei*	Thai NE Env 90; PHLS 216; CDC2721626	Positive (+)
*Burkholderia pseudomallei*	Bp H1406B; CDC0032028	Positive (+)
*Burkholderia pseudomallei*	F1394; 2002721096; 81A442	Positive (+)
*Burkholderia pseudomallei*	CDC2721123; H0929; 98-33; CDC0032024	Positive (+)
*Burkholderia pseudomallei*	Thai NE Human 99; PHLS 392	Positive (+)
*Burkholderia pseudomallei*	CDC1029240; H2001; 2001T-0229	Positive (+)
*Burkholderia pseudomallei*	CDC2721617; PHLS 5; NCTC 8016	Positive (+)
*Burkholderia pseudomallei*	Bp 14; CDC2721618	Positive (+)
*Burkholderia pseudomallei*	Bp H1442; CDC0032025	Positive (+)
*Burkholderia pseudomallei*	MSHR640; CDC8724880	Positive (+)
*Burkholderia pseudomallei*	Australian NT Human 1 97; 465a; CDC8724601	Positive (+)
*Burkholderia pseudomallei*	MSHR99; CDC8724881	Positive (+)
*Burkholderia pseudomallei*	MSHR362; CDC1756207	Positive (+)
*Burkholderia pseudomallei*	MSHR503; CDC8724890	Positive (+)
*Burkholderia pseudomallei*	#711; CDC2721675	Positive (+)
*Burkholderia pseudomallei*	PM19; CDC2734678; 620	Positive (+)
*Burkholderia pseudomallei*	MSHR296; CDC8724908	Positive (+)
*Burkholderia pseudomallei*	MSHR1200; CDC8724883	Positive (+)
*Burkholderia pseudomallei*	CDC2734694; PM40	Positive (+)
*Burkholderia pseudomallei*	PM26; CDC2734683	Positive (+)
*Burkholderia pseudomallei*	Malaysia5 Human; PHLS 75	Positive (+)
*Burkholderia pseudomallei*	MSHR1300; CDC8724901	Positive (+)
*Burkholderia pseudomallei*	PM115; CDC2734709	Positive (+)
*Burkholderia pseudomallei*	STW 424-1; CDC2721825	Positive (+)
*Burkholderia pseudomallei*	Bp40	Positive (+)
*Burkholderia pseudomallei*	MSHR365; CDC8724894	Positive (+)
*Burkholderia pseudomallei*	PM138; CDC2734661; SA923	Positive (+)
*Burkholderia pseudomallei*	Malaysia4 Human; PHLS 79	Positive (+)
*Burkholderia pseudomallei*	BpH1689; CDC0032024	Positive (+)
*Burkholderia pseudomallei*	CDC2721184	Positive (+)
*Burkholderia pseudomallei*	CDC2721634	Positive (+)
*Burkholderia pseudomallei*	CDC1756205	Positive (+)
*Burkholderia pseudomallei*	CDC8724905	Positive (+)
*Burkholderia pseudomallei*	CDC0022203	Positive (+)
*Burkholderia pseudomallei*	CDC2721637	Positive (+)
*Burkholderia pseudomallei*	CDC8724896; 1026b	Positive (+)
*Burkholderia pseudomallei*	CDC8724889	Positive (+)
*Burkholderia pseudomallei*	CDC8724898	Positive (+)
*Burkholderia pseudomallei*	MSHR1655; 2002721686 (*wcbR* mutation)	Negative (−)
*Burkholderia pseudomallei*	CDC8724899	Positive (+)
*Burkholderia pseudomallei*	CDC8724882	Positive (+)
*Burkholderia pseudomallei*	CDC8724900	Positive (+)
*Burkholderia pseudomallei*	CDC8724892	Positive (+)
*Burkholderia pseudomallei*	CDC8724893	Positive (+)
*Burkholderia pseudomallei*	CDC2721761	Positive (+)
*Burkholderia pseudomallei*	CDC8724885	Positive (+)
*Burkholderia pseudomallei*	CDC0022358	Positive (+)
*Burkholderia pseudomallei*	CDC8724877	Positive (+)
*Burkholderia pseudomallei*	CDC1756206	Positive (+)
*Burkholderia pseudomallei*	CDC8724895	Positive (+)
*Burkholderia pseudomallei*	CDC8724903	Positive (+)
*Burkholderia pseudomallei*	CDC8724878	Positive (+)
*Burkholderia mallei*	KC 238; Kweiyang #4; CDC2721277	Positive (+)
*Burkholderia mallei*	Kweiyang #1; CDC2734821	Positive (+)
*Burkholderia mallei*	KC1090; A188 Pasteur Institute; CDC2721278	Positive (+)
*Burkholderia mallei*	India 65-603; CDC0031066	Positive (+)
*Burkholderia mallei*	NCTC 10247; CDC2734315; Turkey 12	Positive (+)
*Burkholderia mallei*	Turkey 1; CDC0031065	Positive (+)
*Burkholderia mallei*	Turkey 5; CDC2734302	Positive (+)
*Burkholderia mallei*	NCTC 10260; CDC2734314; CDC2734301; Turkey 11; GB6; CCUG 19395	Positive (+)
*Burkholderia mallei*	Rob-DASH (2000031281); CDC0031304	Positive (+)
*Burkholderia mallei*	KC 234; 3873; China 7; CDC2721273	Positive (+)
*Burkholderia mallei*	KC 235; 3873-18; CDC2721274	Positive (+)
*Burkholderia mallei*	KC0248; CDC4017733	Positive (+)
*Burkholderia mallei*	KC 1091; A193 Pasteur Institute; CDC2721279	Positive (+)
*Burkholderia mallei*	KC 1092; CDC2721280; 52-236 Pasteur Institute	Positive (+)
*Burkholderia mallei*	BURK011; CDC8724847; C2006251001	Positive (+)
*Burkholderia mallei*	GB9; CDC2734305; Strain 102; NCTC3708	Positive (+)
*Burkholderia mallei*	NCTC 3709 (Strain 106); CDC2724303; GB10	Positive (+)
*Burkholderia mallei*	Turkey 2; BURK063; CDC8724837	Positive (+)
*Burkholderia mallei*	Turkey 3; BURK064; CDC8724838	Positive (+)
*Burkholderia mallei*	Turkey 4; BURK065; CDC8724839	Positive (+)
*Burkholderia mallei*	Turkey 7; BURK068; CDC8724841	Positive (+)
*Burkholderia mallei*	CDC2734300; NCTC10247	Positive (+)
*Burkholderia mallei*	CDC2734301, NCTC10260	Positive (+)
*Burkholderia mallei*	CDC2734317; NCTC3709	Positive (+)
*Burkholderia mallei*	CDC2721275	Negative (−)
*Burkholderia mallei*	CDC2734299	Positive (+)
*Burkholderia mallei*	CDC2734311	Negative (−)
*Burkholderia mallei*	CDC0031063	Positive (+)
*Burkholderia mallei*	CDC0031064	Positive (+)
*Burkholderia mallei*	CDC2721276	Positive (+)
*Burkholderia mallei*	CDC2721648	Positive (+)
*Burkholderia mallei*	CDC2734312	Positive (+)
*Burkholderia mallei*	CDC2721280	Negative (−)
*Burkholderia thailandensis*	CDC3015869 (contains capsule operon)	Positive (+)
*Burkholderia thailandensis*	CDC2721621	Negative (−)
*Burkholderia thailandensis*	CDC2721627	Negative (−)
*Burkholderia thailandensis*	CDC2721121	Negative (−)
*Burkholderia thailandensis*	CDC2721643	Negative (−)
*Burkholderia thailandensis*	CDC2721701	Negative (−)
*Burkholderia thailandensis*	CDC2721723	Negative (−)
*Burkholderia thailandensis*	CDC2721744	Negative (−)
*Burkholderia humptydooensis*	CDC2721687	Negative (−)
*Burkholderia oklahomensis*	CDC4002358	Negative (−)
*Burkholderia oklahomensis*	CDC4021865	Negative (−)
*Burkholderia oklahomensis*	CDC4021866	Negative (−)
*Burkholderia vietnamiensis*	CDC2734483	Negative (−)
*Burkholderia pyrrocinia*	CDC2724646	Negative (−)
*Burkholderia caledonica*	CDC8724197	Negative (−)
*Burkholderia caribensis*	CDC8724200	Negative (−)
*Burkholderia ambifaria*	CDC8724201	Negative (−)
*Burkholderia anthina*	CDC8724199	Negative (−)
*Burkholderia cocovenenans*	CDC2734715	Negative (−)
*Burkholderia ferrariae*	CDC8724209	Negative (−)
*Burkholderia hydrophilia*	CDC2721759	Negative (−)
*Burkholderia fungorum*	CDC8724198	Negative (−)
*Burkholderia glathei*	CDC2734719	Negative (−)
*Burkholderia graminis*	CDC2734716	Negative (−)
*Burkholderia hospita*	CDC8724207	Negative (−)
*Burkholderia kururiensis*	CDC2734717	Negative (−)
*Burkholderia nodosa*	CDC8724205	Negative (−)
*Burkholderia phenazinium*	CDC2734718	Negative (−)
*Burkholderia phenoliruptrix*	CDC8724203	Negative (−)
*Burkholderia phymatum*	CDC8724208	Negative (−)
*Burkholderia phytofirmans*	CDC8724204	Negative (−)
*Burkholderia sacchari*	CDC8724202	Negative (−)
*Burkholderia silvatlantica*	CDC8724206	Negative (−)
*Burkholderia rhizoxinica*	CDC2734772	Negative (−)
*Burkholderia endofungorum*	CDC2734773	Negative (−)
*Burkholderia gladioli*	CDC3027208	Negative (−)
*Escherichia coli*	ATCC 25922	Negative (−)
*Pseudomonas aeruginosa* *	ATCC 27853	Negative (−)
*Streptococcus pneumoniae* *	ATCC 10015	Negative (−)
*Klebsiella pneumoniae* *	ATCC 13883	Negative (−)
*Staphylococcus aureus* *	ATCC 25923	Negative (−)
*Enterobacter cloacae* *	ATCC 23355	Negative (−)
*Providencia stuartii* *	ATCC 33672	Negative (−)

*Indicates strains that were tested for reactivity against mAb 3C5 via western blot.

### Ethics section

Clinical samples from patients with culture-positive melioidosis were obtained from sample archives (no identifiable private information supplied) at Mahidol-Oxford Tropical Medicine Research Unit, Mahidol University, Bangkok, Thailand and Menzies School of Health Research and Northern Territory Clinical School, Royal Darwin Hospital, Darwin, Northern Territory, Australia. Archived and de-identified melioidosis negative serum and urine samples were obtained from the University of Nevada School of Medicine, Reno, NV, USA.

### Quantitation of *B. pseudomallei* in urine samples

*B. pseudomallei* was quantified in urine as previously described [6]. Briefly, 1 µl of urine was plated on Ashdown agar plates and incubated overnight at 37°C [13]. Colonies were counted and expressed as colony forming units (CFU)/ml (Table 1). The remaining urine was centrifuged at 3000 rpm for 5 min. The pellet was then plated on an Ashdown agar plate and incubated overnight. The lower limit of detection was 1 CFU/ml (1 colony from 1 µl) and the upper limit of detection was ≥10^6^ CFU/ml (≥1000 colonies/1 µl). A positive *B. pseudomallei* liquid culture from urine samples that did not show growth on Ashdown agar plates was estimated to contain <10^3^ CFU/ml.

### Monoclonal antibody affinity determination

Antibody-antigen binding experiments were performed using surface plasmon resonance (SPR) with a BIAcore X100 instrument (GE Healthcare, Piscataway, NJ). In each experiment, the running buffer and sample diluent was 1X HBS-EP+ (GE Healthcare): 10 mM HEPES, 150 mM NaCl, 3 mM EDTA, and 0.05% surfactant P20, pH 7.4. Biotinylated CPS was immobilized onto the surface of a streptavidin (SA) sensor chip (GE Healthcare) until 1000 response units (RU) were reached. Purification of CPS has been previously described [10]. A BIAcore flow cell was left unmodified for reference subtraction. To evaluate binding affinity, a two-fold serial dilution of mAb 3C5 (333–5.2 nM) was prepared in HBS-EP+. Each concentration of mAb was injected over the sensor surface at flow rate of 30 µl/min for 60 s, after which mAb was allowed to passively dissociate for 120 s. The sensor surface was regenerated between runs with a 60 s pulse of 4 M MgCl_2_ to ensure the removal of residually bound mAb. The dissociation constant (K_D_) was determined using the steady-state model in BIAevaluation software (GE Healthcare).

### Quantitative antigen-capture ELISA

Detection of CPS by antigen-capture ELISA has been described previously [10]. Briefly, mAb 3C5 (0.25–4 µg/ml) diluted in PBS was incubated overnight at room temperature in 96-well microtiter plates (Immulon 1B, Thermo Scientific). The wells were then washed with PBS-Tween (PBS containing 0.5% Tween 20), and blocked for 90 min in the same solution. Purified CPS in PBS was serially diluted across the 96-well plate from 100–0.006 ng/ml, which was used to generate a standard curve to quantify CPS present in melioidosis patient samples. Wells were washed with PBS-Tween followed by incubation with HRP-labeled mAb 3C5 (2 µg/ml) for 90 min. The wells were then washed and incubated with tetramethylbenzidine substrate (Kirkegaard & Perry Laboratories) for 30 min. Stop solution (1 M H_3_PO_4_) was then added to the wells and the absorbance was read at 450 nm. Patient samples were analyzed by a similar protocol with some minor modifications. Microtiter wells were coated with 2 µg/ml mAb 3C5. Melioidosis patient serum (1∶2 starting dilution) or urine (no starting dilution) was then serially diluted across the microtiter plate. The CPS concentration in urine samples was calculated by applying a linear regression to the plot of log optical density at 450 nm versus log urine dilution with background correction as described by Peterman [14]. An end point optical density of 2-fold over background was used for the calculation of CPS concentrations, using purified CPS as a standard.

### Construction of the AMD LFI

Lateral flow immunoassays were developed using mAb 3C5 targeting the CPS of *B. pseudomallei*. For the test line, 3C5 was sprayed onto a nitrocellulose membrane strip. For the control line goat anti-chicken IgY was sprayed on the same membrane. The conjugate pad contained dried 40 nm gold particles conjugated to mAb 3C5 as well as a small amount of gold conjugated chicken IgY (to react with the control line). The conjugate pad was treated with a borate-based buffer containing a small concentration of detergent and dried for later gold conjugate application. The sample application pad was also treated similarly and dried. The LFI was assembled by combining the sprayed membrane, conjugate pad, and sample pad on top of an adhesive plastic backing. Each layer overlaps by no more than 2–3 mm. Samples were applied to the sample application pad followed by addition of a chase buffer to facilitate capillary action. Certain samples types (e.g. sputum, pus or cultures) were pretreated with a lysis buffer containing low levels of detergents prior to application to the sample pad. LFIs were read after 15 minutes and determined to be positive or negative based on the presence or absence of a pink-red line at the test line in the presence of a positive control line.

### Western blot analysis

A previously described Western blot procedure with semi-dry blotting was used for this study [15]. Briefly, 8×10^6^ bacterial cells were suspended in Laemmli Sample Buffer (Sigma) and boiled for 10 minutes. The samples were run on a 10% SDS gel followed by semi-dry transfer onto a PVDF membrane. mAb 3C5 was used at a final concentration of 0.2 µg/ml. Goat anti-mouse IgG-HRP (Southern Biotech) was used at a 1∶10,000 dilution and signal was detected with a chemiluminescent substrate (Pierce).

### Sample preparation and AMD LFI testing

Bacterial colonies were tested for reactivity on the LFI. An entire single colony was picked with a sterile loop and suspended in two drops of lysis buffer. The entire bacterial suspension was pipetted onto the LFI sample pad followed by the addition of three drops of chase buffer. Three colonies from each bacterial isolate listed in Table 1 were tested in this manner. Culture-proven melioidosis clinical samples (archived) were used to optimize sample preparation. Serum (50 µl) was combined with 150 µl of chase buffer; this solution was then applied to the LFI sample pad. Pus (20 µl) was combined with 100 µl of lysis buffer followed by vortexing. The lysate (20 µl) was then combined with 150 µl of chase buffer and applied to the sample pad. Urine was prepared by first centrifuging a maximum of 10 ml at 3200× *g* for 10 minutes. The supernatant was removed and the pelleted material was suspended in 50 µl of lysis buffer. The lysate (20 µl) was combined with 150 µl of chase buffer and applied to the sample pad. Sputum (50 µl) was combined with 100 µl of lysis buffer followed by vortexing. If the sputum sample was viscous then 20 µl was combined with 150 µl of lysis buffer. The lysate (20 µl) was combined with 150 µl of chase buffer and applied to the sample pad. Pleural fluid (30 µl) was combined with 100 µl of lysis buffer. The lysate (30 µl) was combined with 150 µl of chase buffer and applied to the sample pad. Control serum (50 µl) spiked with purified CPS (five-fold serial dilution) was combined with 150 µl of chase buffer and applied to the AMD LFI. Control urine (50 µl) spiked with purified CPS (five-fold serial dilution) was combined with 150 µl of chase buffer and applied to the AMD LFI. Each test was allowed to flow for 15 min and a digital image was taken of each result.

## Results

Our previous report described the ability of mAb 3C5 to detect *B. pseudomallei* CPS in urine from patients with melioidosis [10]. Although encouraging, further experiments were required before constructing a point-of-care diagnostic assay to determine (i) the affinity of mAb 3C5 for CPS, (ii) the limit of detection of mAb 3C5 for CPS by ELISA, and (iii) the concentration range of CPS that accumulates in melioidosis patient samples.

**Figure 1:**
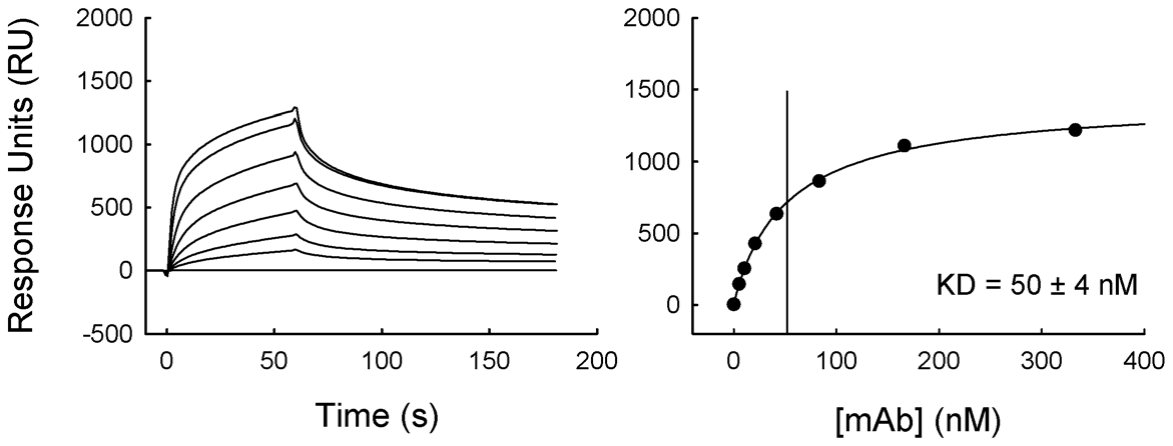
Calculation of mAb 3C5 affinity for CPS. A BIAcore X100 instrument was used to determine the affinity of mAb 3C5 for CPS. Biotinylated CPS was immobilized on the surface of a streptavidin sensor chip. Samples (two-fold serial dilution of mAb 3C5 [333–5.2 nM]) were injected over the sensor surface for 60 s, after which the mAb was allowed to passively dissociate for 120 s (left panel). The dissociation constant (K_D_) was determined using the steady-state model in BIAevaluation software (right panel).

SPR was used to determine the functional affinity of mAb 3C5 for *B. pseudomallei* CPS. Functional affinity is often referred to when describing the collective effects of mAb bivalency and antigen multivalency on binding (since CPS is composed of repeating epitopes). The functional affinity was evaluated on a BIAcore X100 sensor surface coated with immobilized CPS. The binding activity of mAb 3C5 was examined over a 60 s injection pulse. Total (resonance units) RU values were recorded following binding of a series of mAb concentrations (Fig. 1, left panel). These RU values were analyzed using a steady-state binding model (Fig. 1, right panel). This led to the calculation of a 50 nM dissociation constant (K_D_) of mAb 3C5 for CPS. This is a relatively high affinity for a mAb specific to a polysaccharide antigen. This led us to expect that mAb 3C5 would perform well in an antibody-based detection assay.

**Figure 2:**
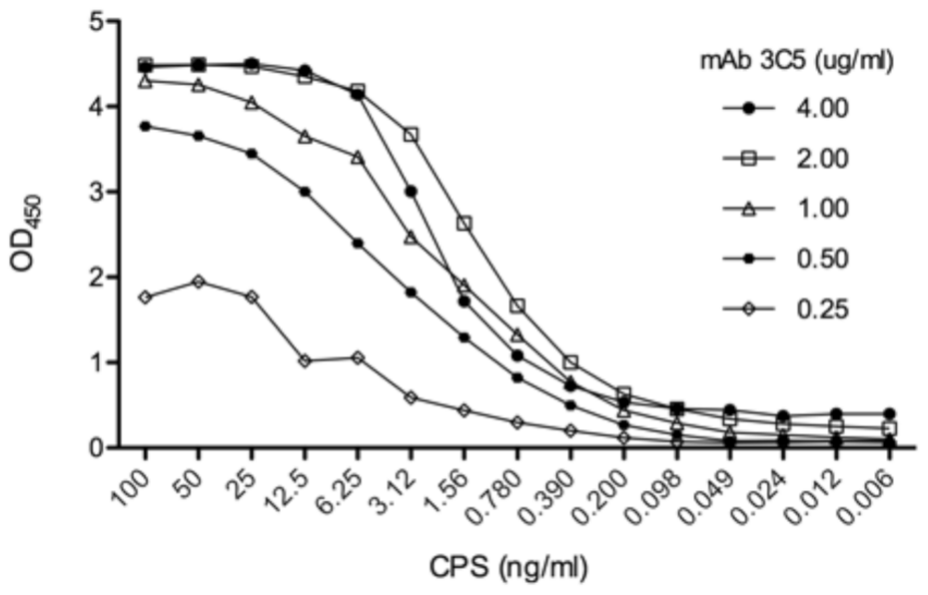
Detection of purified CPS by antigen-capture ELISA. mAb 3C5 was used in the capture phase of the ELISA at the concentrations listed. Following a wash and blocking step, purified CPS was serially diluted across the microtiter plate at the concentrations listed. The wells were then washed and HRP-labeled mAb 3C5 was used in the indicator phase to detect captured CPS. The ELISA was performed in triplicate and mean values are plotted.

An antigen-capture ELISA for CPS [10] was constructed to determine the limit of detection (LOD) that could be achieved with mAb 3C5 (Fig. 2). Due to the polyvalent nature of CPS, mAb 3C5 was used for both capture and detection in this assay. A two-fold serial dilution of mAb 3C5 was incubated in the solid phase of the 96-well microtiter plate vertically across all eight rows. Following a wash and blocking step, a two-fold serial dilution of purified CPS was incubated in the wells (horizontally). Captured CPS was detected with mAb 3C5 labeled with HRP. An optimal LOD of 0.2 ng/ml (2-fold over background) was achieved with a mAb 3C5 coating concentration of 2 µg/ml.

The antigen-capture ELISA was then used to quantify the amount of CPS within serum and urine samples collected from patients with culture-confirmed melioidosis in Thailand. Quantitative cultures were performed on urine samples prior to testing and are reported as CFU/ml (Table 2). Blood cultures were also tested although the CFU/ml was not determined. Each serum (isolated from blood) and urine sample was passed through a 0.22 µm filter in order to remove intact bacterial cells prior to shipment. In our previous report [10] we determined the highest fold dilution of these samples that yielded an ELISA OD_450_ value ≥0.5. For the current study we were able to estimate the concentration of CPS within these samples by comparing the OD values to a standard curve produced with purified CPS (Table 2). CPS was detected in 6/10 filtered urine samples at concentrations ranging from 0.78–448 ng/ml. As expected, the concentration of CPS was higher in samples that contained more CFUs/ml. CPS was detected in all urine samples containing greater than 1.2×10^4^ CFU/ml. CPS was detected in 5/10 filtered serum samples at concentrations ranging from 0.85 to 6.7 ng/ml.

**Table 2:** Quantification of CPS in melioidosis patient serum and urine (filtered) by antigen-capture ELISA.

Urine^a^			Serum		
Sample	CFU/mL^a^	[CPS] (ng/ml)	Sample	Culture result^b^	[CPS] (ng/ml)
UID1	2.3×10^4^	2.7	MSID1	+	5.4
UID2	>1×10^5^	448	MSID2	+	<LOD
UID3	7.5×10^4^	20	MSID3	+	6.7
UID4	1.2×10^4^	0.78	MSID4	+	3.3
UID5	>1×10^5^	66	MSID5	+	<LOD
UID6	3.5×10^3^	<LOD^c^	MSID6	+	0.85
UID7	>1×10^5^	187	MSID7	+	<LOD
UID9	<1×10^3^	<LOD	MSID8	+	<LOD
UID10	∼1×10^3^	<LOD	MSID9	+	1.6
UID12	∼1×10^3^	<LOD	MSID10	+	<LOD

a Serum and urine were collected from different patients.

b Blood cultures (serum) are reported only as positive or negative.

c CPS concentrations of these samples were below the LOD of the ELISA.

**Figure 3:**
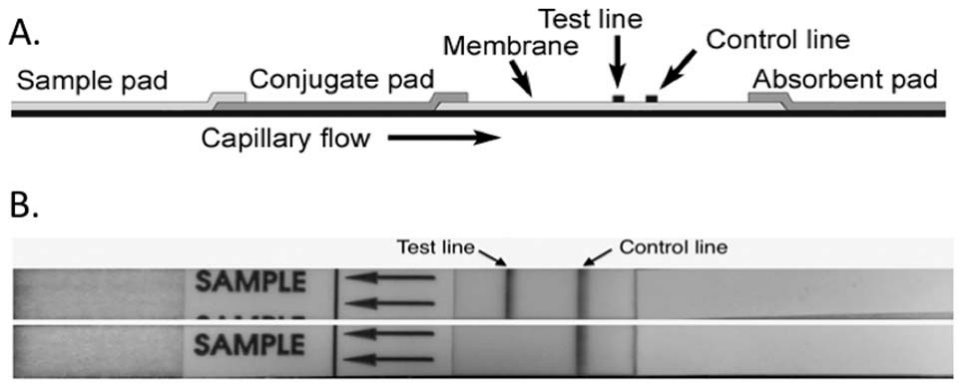
Prototype Active Melioidosis Detect (AMD) LFI. (A) Schematic of LFI components. (B) *B. pseudomallei* strain Bp82 colony grown on an agar plate was picked and suspended in 2 drops of lysis buffer. The lysate was added to the sample pad followed by three drops of LFI chase buffer (top LFI). The LFI was imaged following a 15 min run time. The same test condition were used with a colony of *E. coli* (bottom LFI).

Following successful detection of CPS by ELISA, a prototype AMD LFI was constructed. A schematic of the components of the LFI is depicted in Fig. 3A. Initial LFI testing was performed on *B. pseudomallei* strain Bp82, a select agent excluded strain [16], and a strain of *E. coli* (negative control). Bp82 was not included in Table 1 since the strain was derived from *B. pseudomallei* strain 1026b, which is listed in Table 1. For each test, one single colony was collected with a sterile loop and resuspended in two drops of lysis buffer. The lysate was pipetted onto the LFI sample pad followed by addition of three drops of chase buffer. The fluid migrates by capillary action into the conjugate pad where gold-labeled mAb 3C5 binds to CPS present in the lysate. The gold-labeled mAb 3C5/CPS complex then migrates into the nitrocellulose membrane and is captured at the test line, which is unlabeled mAb 3C5 bound to the membrane. The absorbent or wicking pad allows for efficient capillary flow of the sample across the test line. The LFI used to analyze Bp82 showed test line and control line reactivity (Fig. 3B, top LFI) while the *E. coli* LFI was reactive only on the control line (Fig. 3B, bottom LFI). The tests are run for 15 minutes and results are recorded and imaged. Presence of the LFI control line ensures the test has run properly.

The LFI was tested for reactivity to *B. pseudomallei* and *B. mallei* in addition to other near neighbor species (Table 2). Strain panels tested included isolates selected by the Stakeholder Panel on Agent Detection Assay (SPADA) *Burkholderia* Working Group. The SPADA *Burkholderia* panel was compiled by a number of key stakeholders from federal agencies and biothreat researchers [17]. *B. mallei* has recently been shown to produce the identical manno-heptose capsule as *B. pseudomallei* [18]; we have previously shown mAb 3C5 reactivity to *B. mallei* CPS by Western blot [10]. The LFI testing was performed at the Centers for Disease Control and Prevention to evaluate analytical reactivity and specificity on inclusivity and exclusivity strain panels. Three colonies from each isolate listed in Table 1 were tested separately on the LFI. Of the *B. pseudomallei* isolates tested, 76/77 (98.7%) were positive; 30/33 (90.9%) of the *B. mallei* isolates were also positive. In addition, 35/36 (97.2%) of near neighbor species were negative by LFI. Eight *Burkholderia thailandensis* isolates were tested, and seven were negative. Other near neighbor species where also tested, including *Burkholderia humptydooensis sp. nov.*, *Burkholderia oklahomensis* and *Burkholderia* cepacia complex (Bcc) species, all of which were negative. In addition, other medically relevant species of bacteria were negative for reactivity by Western blot (see footnote to Table 1) to mAb 3C5 (data not shown).

**Figure 4:**
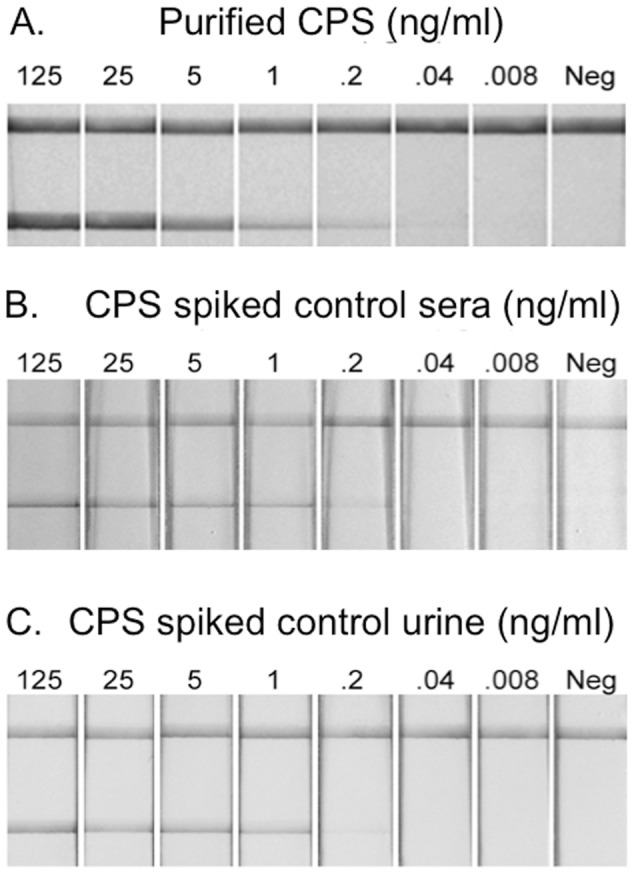
Determination of the LOD of the AMD LFI. (A) Purified CPS was diluted in chase buffer at the indicated concentration and applied to the LFI sample pad. Results were photographed after 15 min. Purified CPS was also diluted in human control sera (B) and human control urine (C).

The LOD of the AMD LFI was determined to verify that the analytical sensitivity of the assay was sufficiently low to be used to detect CPS in patient samples. Purified CPS was tested on the LFI to determine the LOD under optimal conditions (Fig. 4). Dilutions of CPS were prepared in chase buffer and applied to the LFI sample pad. The LOD was estimated at or slightly below 0.2 ng/ml. In addition, purified CPS was spiked into control serum (Fig. 4B) and urine (Fig. 4C). Under these conditions the LOD was increased slightly when compared to dilution in chase buffer alone, however a clear reaction was apparent at 0.2 ng/ml.

**Figure 5:**
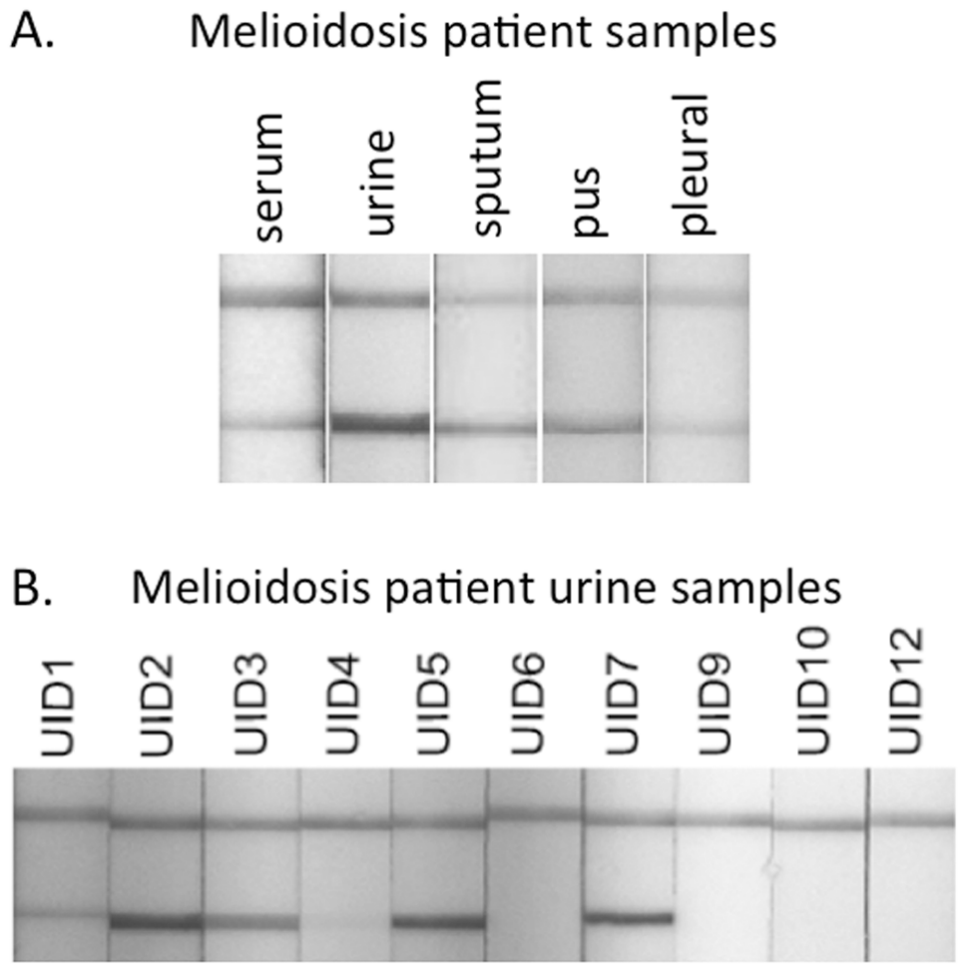
Prototype AMD LFI for detection of *B. pseudomallei* CPS in melioidosis patient samples. (A) Preliminary testing of a variety of archived patient samples from Australia and Thailand. (B) Detection of CPS in melioidosis patient urine samples (filtered) listed in Table 2. Urine (50 µl) was combined with 100 µl of chase buffer and applied to the sample pad. Note that samples that were positive by antigen-capture immunoassay (Table 2) were also positive by LFI and the levels of CPS detected between both assays are congruent.

The ability of the AMD LFI to accept a variety of patient samples was assessed with a limited number of culture-positive melioidosis samples in Australia. These samples were also used to optimize sample preparation for the AMD LFI. The LFI was designed to accept multiple sample matrices, which is critically important for the diagnosis of melioidosis. As shown in Fig. 5A the samples tested included serum, urine, sputum, pus and pleural fluid collected from culture-confirmed melioidosis patients (samples were not collected from the same patient) in Thailand and Australia. Preparation of each sample prior to application to the sample pad is described in the Methods section. The melioidosis patient urine samples that were tested by antigen-capture ELISA (Table 2) were also tested by LFI (Fig. 5B). The urine samples that were positive by ELISA were also positive by LFI. Qualitatively, the test line intensity of the positive urine samples was congruent with their corresponding ELISA values.

## Discussion

A number of assays have been developed to diagnose melioidosis prior to culture results becoming available. PCR has been developed but is not in routine practice because it is limited by low sensitivity, most likely stemming from the low concentration of *B. pseudomallei* in blood and the co-purification of PCR inhibitors with target DNA [19]–[21]. However, a recently developed Type III secretion system (TTS-1) real-time PCR assay has been shown to be superior to previously developed PCR assays for detection of *B. pseudomallei* DNA in clinical specimens [21]. When compared to culture the TTS-1 assay had a sensitivity and specificity of 80% and 100%, respectively. The indirect hemagglutination assay (IHA) is a rapid and inexpensive method used to detect antibodies produced during infection that are specific to *B. pseudomallei*. However, a large percentage of healthy individuals in endemic areas are seropositive [22], [23]. This point is underscored by the fact that nearly 70% of children in northeast Thailand are seropositive for *B. pseudomallei* antigens [24], [25]. Consequently, the IHA (or any serological test for melioidosis) has limited clinical utility in the endemic setting [1], [26].

Antigen detection by immunofluorescence assay (IFA) or latex agglutination is commonly used in endemic areas. IFA is used in northeast Thailand for rapid diagnosis directly from patient samples containing high levels of *B. pseudomallei* (sputum, pus, urine and respiratory secretions) [27], [28] and from blood cultures [29]. The main drawback of IFA is the requirement for a fluorescent microscope and the requisite expertise, which is not feasible in most endemic settings. In addition, although specificity of the IFA is high, the sensitivity has recently been determined to range from 45–48% when used directly on clinical samples [27]. Latex agglutination is an inexpensive technique that is effective at identifying *B. pseudomallei* from cultures of patient samples grown on agar plates or within liquid broth [30]–[33]. The agglutination assay is able to detect *B. pseudomallei* at concentrations of 1–2×10^6^ CFU/ml; this limits its utility to cultured patient samples or colonies isolated on solid agar [32], [33].

Our LFI is similar in design to those currently used for the diagnosis of *Streptococcus pneumoniae* and *Legionella pneumophila* [34]. The *L. pneumophila* assay is a first-line test that relies on detection of antigen produced by the bacterium within patient urine [35]. We anticipate the AMD LFI can also be used as a first-line test and offer an improvement over the current rapid techniques for the diagnosis of melioidosis. In addition we believe lateral flow devices are well suited for resource poor settings in that they are inexpensive, rapid, sensitive, and stable at room temperature. In addition, LFIs do not require expensive equipment and they can accept multiple sample matrices, two characteristics that are essential for the diagnosis of melioidosis in resource poor settings.

IgG3 mAb 3C5 possesses many important characteristics that are necessary for the development of an antigen detection assay. It has a relatively high affinity for its target antigen and shows acceptable analytical reactivity and specificity. The high affinity translates into a lower limit of detection for CPS by ELISA and LFI. Interestingly, the LFI had a comparable analytical sensitivity to the ELISA (∼0.2 ng/ml) when CPS was diluted in chase buffer. The analytical sensitivity was slightly lower when CPS was spiked into control serum and urine. When tested by LFI, 98.7% of *B. pseudomallei* isolates were positive while 97.2% of near neighbor species were negative. Both the false-negative and false-positive LFI results can be explained through sequencing analysis. The one isolate that produced a false negative (MSHR1655) originated from a patient that developed a persistent asymptomatic *B. pseudomallei* infection in Australia. A frameshift mutation was identified within the *wcbR* gene of this isolate [36]. A *B. pseudomallei* strain (K96243) with a *wcbR* mutation was recently shown to have greatly reduced CPS expression [37]. The one *B. thailandensis* isolate that produced the false positive had been previously shown to encode the CPS biosynthetic operon [38], [39].

An essential aspect of the current study was the quantification of CPS within patient samples. This was accomplished by comparing ELISA values generated from patient samples with a standard curve generated with known concentrations of purified CPS. Over half of the filtered serum and urine samples from melioidosis patients had levels of CPS within the detection range of the AMD LFI. The LFI detected CPS in 6/10 culture-positive urine samples from melioidosis patients. We anticipate that if the urine had not been filtered more of the samples would have been positive. Patient serum samples were not tested on the LFI due to insufficient volumes, but half contained concentrations of CPS (as determined by ELISA) that could be detected by the AMD LFI. This is encouraging since the mean concentration of *B. pseudomallei* in patient blood is ∼1 CFU/ml [5], [6]. We anticipate that CPS may be shed from internal abscesses into the blood; so theoretically, even if the concentration of bacteria in blood is low, the concentration of CPS may be within the detectable range of the LFI. CPS could not be detected in filtered urine samples that contain low levels of bacteria, suggesting that CPS may not be shed into urine to detectable levels from the blood.

This study describes the development and optimization of a prototype LFI for the rapid diagnosis of melioidosis, including protocols for the preparation of different sample types. This is essential since the LFI will be used to test at least four different bodily fluids, bacterial colonies grown on solid agar, and bacterial liquid cultures from patient samples. We anticipate routine testing can be performed on all patient sample types, and the clinical sensitivity of the LFI will be related to the specific sample type tested. The sample type producing the lowest sensitivity will most likely be blood; this is related to the low levels of *B. pseudomallei* found in this sample type [5], [6]. However, we believe when the LFI is used to test urine, sputum, and pus, high sensitivity will be achieved due to the increased CFU/ml values in these matrices. Now that we have developed reliable sample preparation guidelines we will perform a larger preclinical analysis in the endemic areas of Thailand and Australia. The preclinical analysis will compare the performance of the LFI with the TTS-1 real-time PCR assay, IFA, and culture (the current “gold standard” for diagnosis of melioidosis). This will allow us to determine clinical sensitivity and specificity and the diagnostic utility of the assay. Further studies are underway to isolate additional CPS specific mAbs that possess higher affinities than 3C5. Incorporation of such mAbs into the AMD LFI may lead to increased analytical and clinical sensitivity.
